# Noninvasive prediction of severe histopathology in drug-induced liver injury using a dual elastography-based machine learning model

**DOI:** 10.1007/s00330-026-12644-y

**Published:** 2026-06-08

**Authors:** Luping Qiu, Liyun Xue, Hui Feng, Fankun Meng, Ying Zheng, Guangwen Cheng, Yao Zhang, Zhiyong Yin, Jing Wu, Jiabao Zhu, Jing Liang, Jie Yu, Ping Liang, Hong Ding

**Affiliations:** 1 Department of Ultrasound, Huashan Hospital, Fudan University, Shanghai, China; 2 Department of Interventional Ultrasound, Fifth Medical Center of Chinese PLA General Hospital, Beijing, China; 3 Department of Ultrasound, Beijing Youan Hospital, Capital Medical University, Beijing Institute of Hepatology, Beijing, China; 4 Department of Ultrasound, Beijing Ditan Hospital, Capital Medical University, Beijing, China; 5 Department of Ultrasound, Nantong Third Hospital Affiliated to Nantong University, Nantong, China

**Keywords:** Chemical and drug-induced liver injury, Elasticity imaging techniques, Machine learning, Severity of illness index, Risk assessment

## Abstract

**Objectives:**

To develop and validate a machine learning (ML) model integrating dual elastography, clinical features, and serum biomarkers for noninvasive prediction of severe drug-induced liver injury (DILI).

**Materials and methods:**

This prospective multicenter study enrolled consecutive DILI patients undergoing liver biopsy and dual elastography. Severe DILI was defined as Scheuer inflammation grade plus fibrosis stage ≥ 5 (G + S ≥ 5). Dual elastography-derived activity index (A index) and fibrosis index (F index) correlated with pathological inflammation (G0–4) and fibrosis (S0–4) stages. The dataset was stratified and split 7:3 into training and test sets. LASSO regression was applied for feature selection. Eight ML models were constructed and compared, optimized using 5-fold cross-validation and Bayesian methods. Performance was evaluated by area under the curve (AUC), sensitivity, and specificity. SHapley Additive exPlanations (SHAP) were used to interpret the models.

**Results:**

A total of 305 participants were included (median age 49 years, IQR 40–56; 98 male), comprising 55 with severe DILI and 250 without. A and F indices increased with inflammation grade and fibrosis stage, respectively (*p* < 0.01). Combining clinical and dual elastography features with serum biomarkers, the optimized regularized regression model performed best in the test set (AUC 0.862 [95% CI: 0.776–0.947]; sensitivity 81.2%; specificity 74.7%). SHAP analysis identified the dual elastography indices collectively as the dominant predictors. An online risk calculator was developed from this model.

**Conclusion:**

We developed an explainable, high-performing and dual elastography-based ML model to predict severe DILI, facilitating risk stratification and preliminary management.

**Key Points:**

***Question*** *Severe drug-induced liver injury (DILI) leads to a poor prognosis, yet early noninvasive identification remains challenging due to non-specific serum markers and invasive biopsy.*

***Findings*** *The regularized regression model integrating dual elastography, clinical features and serum biomarkers, non-invasively and robustly predicted severe histologic injury in DILI.*

***Clinical relevance*** *This dual elastography-based machine learning model offers a noninvasive solution for the early identification of DILI patients with severe tissue injury, minimizing unnecessary biopsy and improving prognosis. Clinicians can utilize the online tool for real-time risk stratification at:* https://wznng666.shinyapps.io/RR55555/.

**Graphical Abstract:**

**Figure:**
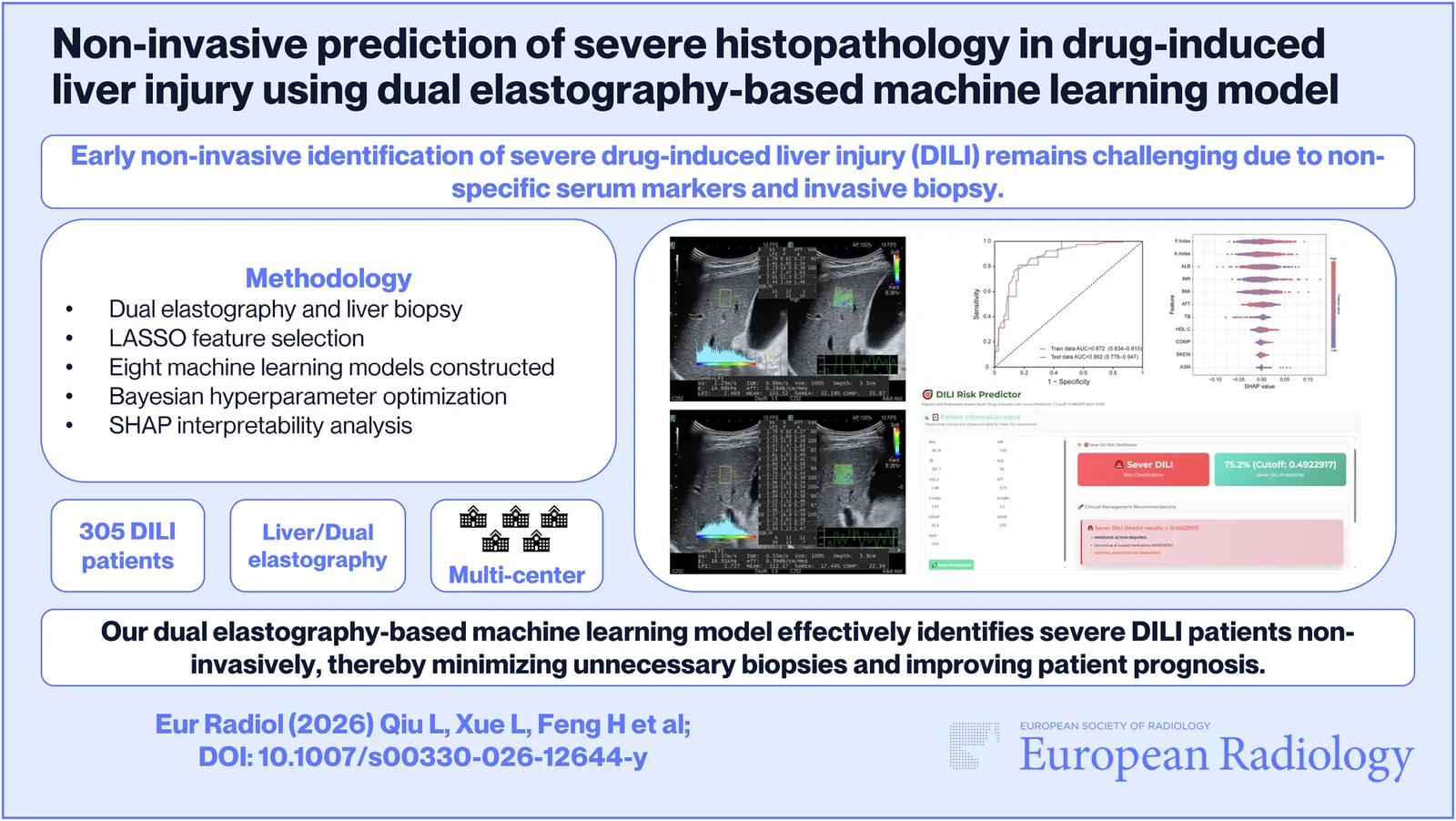

## Introduction

Drug-induced liver injury (DILI) is a challenging disorder with a broad clinical spectrum [1–3]. Although most patients recover spontaneously after drug withdrawal, approximately 20% progress to acute liver failure or cirrhosis [4–6]. Histopathologically, severe cases during the active phase are characterized by the coexistence of intense inflammation and fibrosis [7]. While fibrosis typically develops secondary to drug-induced hepatocellular necrosis, preexisting fibrosis at DILI onset dictates worse outcomes. Importantly, both inflammation grade and fibrosis stage predict adverse outcomes, as isolating either dimension incompletely captures the overall injury burden [8]. Consequently, accurately assessing tissue injury severity in DILI is essential for early risk stratification and optimizing management.

Current DILI severity evaluation methods remain suboptimal. Conventional serological markers lack specificity and are affected by non-hepatic factors [9]. Traditional scoring systems, such as Hy’s law and the model for end-stage liver disease (MELD), offer limited prognostic value [10]. While composite models (e.g., BNR-6, Ashby recovery score) and novel biomarkers (e.g., cytokeratin-18, osteopontin) have been explored, they remain insufficiently predictive and lack standardized assay thresholds, respectively [11–13]. Although liver biopsy is the reference standard for assessing severity, its invasiveness precludes routine use [14].

Imaging modalities overcome the spatial limitations of serological tests, allowing visualization of hepatic structures. Although MRI has shown promise in identifying features and predicting DILI chronicity, widespread use is limited by high costs and complex workflows [15, 16]. Shear wave elastography (SWE) has been extensively validated for liver fibrosis assessment; however, stiffness measurements are confounded by inflammation and congestion [17, 18]. This limitation is critical in DILI, where inflammation and fibrosis require concurrent evaluation [13]. Consequently, SWE cannot accurately reflect fibrotic burden or independently assess inflammatory activity.

Emerging dual elastography, integrating SWE and real-time tissue elastography (RTE), offers a solution to this dilemma. It simultaneously obtains the fibrosis index (F index), reflecting tissue stiffness, and the activity index (A index), characterizing tissue viscoelasticity, enabling quantitative evaluation of fibrosis and inflammation [19]. Although this approach is valuable in chronic hepatitis B and metabolic dysfunction-associated steatotic liver disease (MASLD) for differentiating inflammation and fibrosis stages, its application in DILI remains unexplored [20–22]. This prospective study aims to: (1) elucidate correlations between dual elastography parameters and histopathological severity in DILI; and (2) develop and validate a dual elastography-based machine learning (ML) model for noninvasive identification of severe cases, defined as inflammation grade plus fibrosis stage ≥ 5 (G + S ≥ 5). Addressing this unmet need may facilitate early risk stratification, timely intervention, and improved outcomes for high-risk DILI patients.

## Materials and methods

### Study design and participants

The prospective study adhered to the 2013 Declaration of Helsinki ethical principles, was approved by institutional ethics committees, and registered at ClinicalTrials.gov (NCT04625166); all participants provided written informed consent.

Between December 2020 and June 2022, the study was conducted across five tertiary hospitals in China to recruit consecutive patients suspected of DILI. Inclusion criteria included: age 18–80 years; Roussel Uclaf causality assessment method (RUCAM) score ≥ 6 or 3–5 with expert consensus confirmation [23]; completion of liver biopsy and dual elastography within 3 days; and informed consent. Exclusion criteria included: concurrent liver diseases with other established etiologies (viral hepatitis, alcoholic liver disease, autoimmune hepatitis, genetic/metabolic liver diseases, primary cholestatic liver disease, or parasitic infections); inadequate liver biopsy sample; failed dual elastography; and pregnancy.

### Data collection

All patients underwent dual elastography within 3 days before liver biopsy. Demographic characteristics, medication history, and peak biochemical indicators in the acute phase of DILI were collected.

### Dual elastography examination

Trained sonographers performed all examinations using a Hitachi Arietta 850 ultrasound system (FUJIFILM Healthcare) with a C252 transducer (1–6 MHz). With the patient in the supine position, a sampling box was placed 1–2 cm beneath the right hepatic capsule, avoiding large vessels. Standardized dual elastography mode measurements required a reliability index (VsN) ≥ 60%; ten measurements were taken per patient, with the median value used for analysis. The diagnostic performance of the A index and F index for differentiating histopathological grades and stages was evaluated using the receiver operating characteristic (ROC) curve analysis. The optimal cutoff values were determined by the Youden index. Area under the curve (AUC), sensitivity, specificity, and 95% confidence intervals (CI) were calculated.

The software provided 14 quantitative parameters: 11 tissue dispersion parameters (MEAN [mean relative strain value], SD [standard deviation], %AREA [area of low strain], COMP [complexity of low strain area], KURT [kurtosis], SKEW [skewness], CONT [contrast], ENT [entropy], IDM [inverse difference moment], ASM [angular second moment], and CORR [correlation]), two shear wave parameters (Shear wave velocity [Vs] and Young’s modulus [E]), and the acoustic attenuation coefficient (ATT). Three derived indicators were calculated: F index, A index, and Liver Fibrosis Index (LFI) (formulas are provided in the Supplementary Material).

### Liver biopsy and pathologic histologic examination

All patients underwent ultrasound-guided percutaneous right lobe liver biopsy within 3 days following dual elastography. Pathological sections from all centers were reviewed by two liver pathologists (≥ 10 years of experience), blinded to clinical data and dual elastography results. Liver injury severity was assessed using the semi-quantitative histological Scheuer scoring system, which independently evaluates two dimensions: Grade (G0–G4), reflecting the degree of inflammatory activity (ranging from absent to severe interface hepatitis with bridging necrosis), and Stage (S0–S4), reflecting the extent of fibrosis (ranging from no fibrosis to cirrhosis). Higher scores indicate greater histological severity [24]. Disagreements were resolved by consensus, and the results served as the reference standard.

### Data preparation and feature selection

The primary outcome was histologically defined severe DILI (Scheuer G + S ≥ 5) [25]. Based on clinical consensus and literature review, 20 ultrasound parameters (14 dual elastography metrics, three derived indicators, portal vein diameter (PVD), portal vein velocity (PVV), and depth) and 17 clinical features were selected as candidate predictors. Outliers detected by the interquartile range (IQR) method were reviewed and either corrected or excluded based on clinical validation. Missing values were imputed using random forest multiple imputation (maximum missing rate per variable < 7%). The dataset was randomly split 7:3 into training (*n* = 214) and internal test (*n* = 91) sets via stratified sampling, preserving the original severe DILI prevalence and ensuring representative class distributions.

Feature selection was performed on the training set using a two-step procedure. First, the Least Absolute Shrinkage and Selection Operator (LASSO) logistic regression was applied to the initial candidate variables. The optimal penalty parameter was determined via 5-fold cross-validation (CV) based on the minimum mean CV error. Second, the LASSO-selected features were combined with two pre-defined core parameters (F index and A index), and collinearity within this candidate set was assessed using Pearson correlation coefficients. To avoid information redundancy, variables exhibiting extremely high correlation (|r| > 0.9) were excluded, with priority given to retaining the pre-defined core parameters.

### Model construction and evaluation

The Synthetic Minority Oversampling Technique (SMOTE) was applied to the training set to address class imbalance [26], using k = 5 nearest neighbors and an over_ratio = 0.9. Eight ML models were constructed: Random Forest (RF), Support Vector Machine (SVM), Generalized Linear Model (GLM), Gradient Boosting Machine (GBM), K-Nearest Neighbors (KNN), Artificial Neural Network (NNET), Regularized Regression (RR), and Decision Tree (DT). Initial hyperparameter tuning used 5-fold CV with grid search, and optimized parameters are provided in Supplementary Table S1. The best-performing model then underwent further fine-tuning using Bayesian optimization with 5-fold CV. Model performance was evaluated using accuracy, precision, recall (sensitivity), specificity, F1-score, and AUC with 95% CI. Interpretability was assessed using SHapley Additive exPlanations (SHAP) to quantify feature contributions [27]. Finally, the model was deployed as an online predictive calculator with the threshold determined by the Youden index.

### Statistical analysis

Continuous variables were assessed for normality via the Shapiro–Wilk test; normally distributed variables are presented as mean ± SD and compared using the *t*-test, while non-normally distributed variables are presented as median (IQR) and compared using the Wilcoxon rank-sum test. Categorical variables are presented as frequency (percentage) and compared using the chi-square test or Fisher’s exact test (for expected frequencies < 5). All analyses were performed in R (v4.3.2) with a fixed random seed for reproducibility, using ‘glmnet,’ ‘caret,’ ‘tidymodels,’ ‘themis,’ ‘fastshap,’ and ‘shapviz’ for model construction, evaluation, and interpretation. A two-sided *p*-value < 0.05 was considered statistically significant.

## Results

### Participant characteristics

**Fig. 1:**
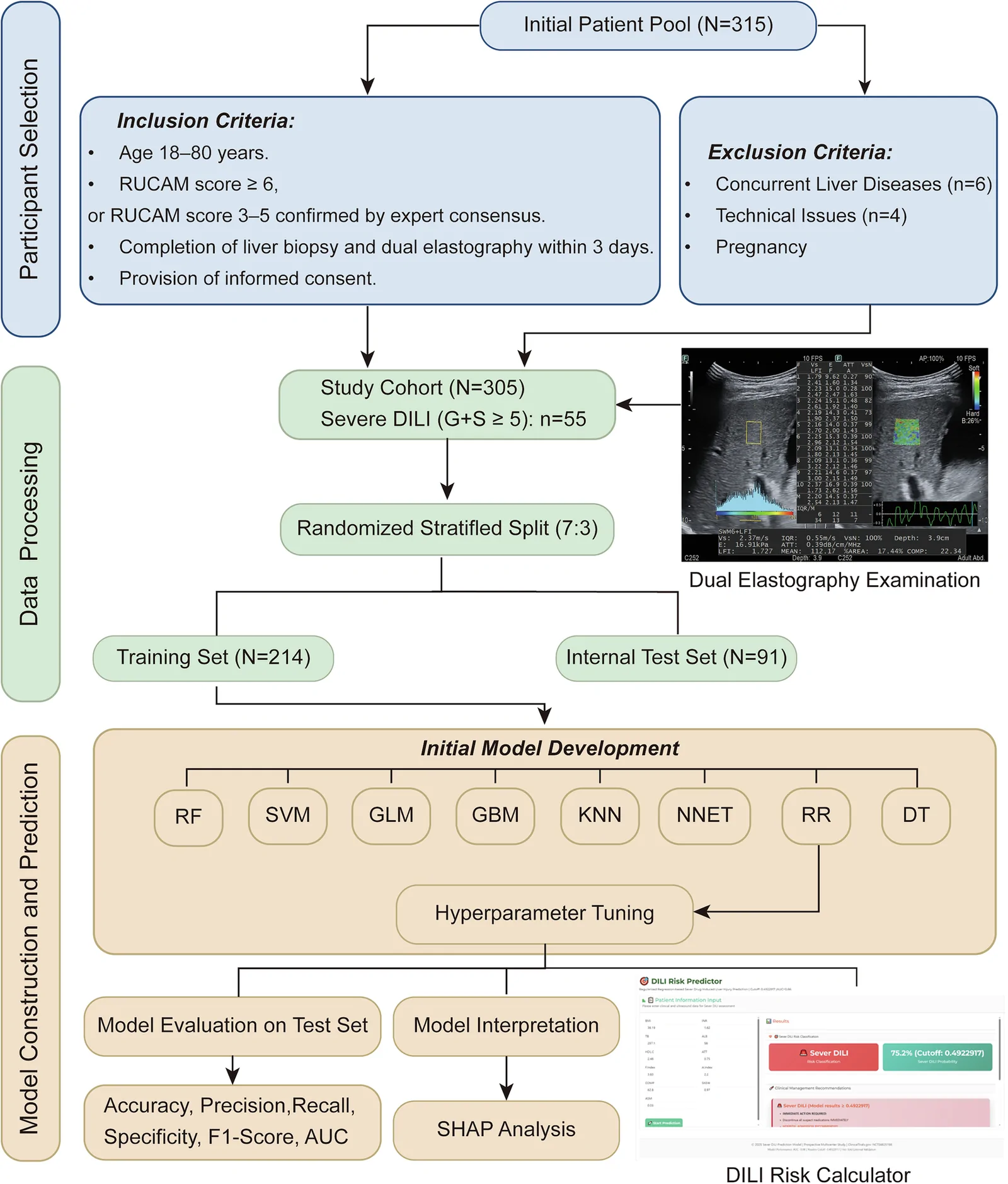
Flowchart of the development and validation of a machine learning model for predicting severe DILI. RUCAM, Roussel Uclaf causality assessment method; DILI, drug-induced liver injury; RF, random forest; SVM, support vector machine; GLM, generalized linear model; GBM, gradient boosting machine; KNN, K-nearest neighbors; NNET, artificial neural network; RR, regularized regression; DT, decision tree; SHAP, SHapley Additive exPlanations

The research process is shown in Fig. 1. Of 305 DILI patients included, 55 (18.0%) reached the composite endpoint (G + S ≥ 5) and were classified as severe DILI. Baseline characteristics are detailed in Table 1. Compared with the mild-to-moderate group (G + S < 5, *n* = 250), the severe group was older, with higher BMI and INR, but lower ALB (all *p* < 0.01). Regarding dual elastography, the severe group showed significantly higher A index, F index, Vs, E, %AREA, COMP, SD, SKEW, and IDM, but lower MEAN and ATT. For model development, the cohort was randomly split 7:3 with stratification into a training set (*n* = 214) and an internal test set (*n* = 91). Stratified sampling successfully preserved the prevalence of severe DILI (18.2% vs. 17.6%, *p* = 1), and all baseline characteristics were comparable between the two sets (Supplementary Table S2).

**Table 1:** Comparison of baseline characteristics between severe and non-severe DILI patients

	Overall (305)	G + S < 5 (250)	G + S ≥ 5 (55)	*p*-value
Age	49.00 [40.00, 56.00]	47.50 [38.00, 55.00]	54.00 [43.00, 59.00]	0.003
Female	207 (67.9)	168 (67.2)	39 (70.9)	0.709
BMI	23.04 [21.04, 25.00]	22.76 [20.77, 24.59]	23.83 [22.72, 27.02]	0.003
INR	1.04 [0.98, 1.11]	1.03 [0.97, 1.09]	1.12 [1.06, 1.19]	< 0.001
TB	23.40 [14.60, 50.90]	22.80 [14.22, 53.12]	25.40 [17.35, 45.10]	0.582
ALT	73.00 [39.00, 151.00]	76.00 [41.00, 156.50]	61.00 [34.00, 142.50]	0.269
AST	59.00 [36.00, 142.00]	59.00 [36.00, 124.75]	61.00 [39.00, 184.00]	0.417
GGT	103.00 [55.00, 220.00]	101.50 [53.25, 220.50]	119.00 [64.00, 209.00]	0.284
ALB	37.50 [34.30, 41.00]	38.00 [35.00, 41.15]	35.00 [32.00, 37.00]	< 0.001
ALP	110.00 [84.00, 146.00]	109.00 [83.25, 148.00]	115.00 [89.50, 133.50]	0.934
Eosinophils	2.10 [1.00, 3.20]	2.10 [1.00, 3.18]	1.90 [1.10, 3.25]	0.836
SCr	59.00 [49.00, 70.00]	60.00 [49.00, 71.00]	58.00 [49.00, 68.00]	0.507
BUN	3.80 [3.10, 4.90]	3.85 [3.10, 4.90]	3.77 [3.12, 4.91]	0.867
TG	1.49 [1.05, 2.12]	1.50 [1.06, 2.14]	1.40 [0.86, 1.84]	0.255
TC	4.00 [3.40, 4.86]	4.00 [3.40, 4.89]	3.99 [3.42, 4.62]	0.281
HDL.C	0.97 [0.71, 1.26]	0.95 [0.68, 1.26]	1.11 [0.84, 1.34]	0.099
LDL.C	2.70 [2.20, 3.30]	2.71 [2.22, 3.40]	2.60 [2.10, 3.14]	0.111
G				< 0.001
1	81 (26.6)	81 (32.4)	0 (0.0)	
2	95 (31.1)	88 (35.2)	7 (12.7)	
3	118 (38.7)	74 (29.6)	44 (80.0)	
4	11 (3.6)	7 (2.8)	4 (7.3)	
S				
0	145 (47.5)	145 (58.0)	0 (0.0)	< 0.001
1	82 (26.9)	80 (32.0)	2 (3.6)	
2	54 (17.7)	24 (9.6)	30 (54.5)	
3	20 (6.6)	1 (0.4)	19 (34.5)	
4	4 (1.3)	0 (0.0)	4 (7.3)	
PVD	10.80 [10.00, 11.30]	10.85 [10.00, 11.28]	10.72 [10.00, 11.40]	0.911
PVV	22.60 [20.00, 26.20]	22.65 [20.22, 26.00]	22.00 [19.00, 27.20]	0.686
MEAN	109.00 [99.40, 114.00]	109.50 [102.00, 114.00]	101.00 [93.50, 111.00]	< 0.001
SD	59.81 (9.74)	58.88 (9.57)	64.07 (9.43)	< 0.001
%AREA	22.30 [14.60, 32.50]	21.30 [14.20, 30.08]	31.10 [17.60, 39.60]	< 0.001
COMP	25.50 [21.80, 30.70]	24.66 [21.70, 28.98]	29.10 [23.60, 35.70]	0.001
KURT	2.38 [2.26, 2.58]	2.39 [2.28, 2.59]	2.37 [2.23, 2.51]	0.195
SKEW	0.26 [0.13, 0.50]	0.25 [0.12, 0.46]	0.37 [0.24, 0.58]	0.001
CONT	293.00 [232.00, 345.00]	288.50 [227.50, 341.00]	313.00 [252.00, 357.00]	0.022
ENT	3.69 [3.64, 3.72]	3.69 [3.64, 3.73]	3.69 [3.61, 3.72]	0.13
IDM	0.10 [0.09, 0.11]	0.09 [0.09, 0.11]	0.10 [0.09, 0.13]	0.049
ASM	0.00 [0.00, 0.00]	0.00 [0.00, 0.00]	0.00 [0.00, 0.00]	0.179
CORR	0.96 [0.95, 0.96]	0.96 [0.95, 0.96]	0.96 [0.95, 0.96]	0.53
Depth	3.80 [3.50, 4.20]	3.80 [3.50, 4.20]	3.80 [3.55, 4.30]	0.27
ATT	0.53 [0.46, 0.60]	0.54 [0.46, 0.61]	0.49 [0.43, 0.56]	0.003
LFI	1.92 [1.49, 2.55]	1.85 [1.44, 2.42]	2.48 [1.63, 2.92]	0.001
Vs	1.82 [1.54, 2.16]	1.77 [1.48, 2.08]	2.18 [1.94, 2.49]	< 0.001
E	9.95 [7.15, 14.05]	9.39 [6.61, 13.04]	14.32 [11.21, 18.62]	< 0.001
F index	1.78 [1.33, 2.22]	1.65 [1.26, 2.12]	2.18 [1.85, 2.62]	< 0.001
A index	1.28 (0.33)	1.23 (0.32)	1.50 (0.29)	< 0.001

Data are presented as median (25%, 75%) for continuous variables and number (percent) for categorical variables

*BMI* body mass index, *INR* international normalized ratio, *TB* total bilirubin, *ALT* alanine aminotransferase, *AST* aspartate aminotransferase, *GGT* γ-glutamyl transferase, *ALB* albumin, *ALP* alkaline phosphatase, *SCr* serum creatinine, *BUN* blood urea nitrogen, *TG* triglycerides, *TC* total cholesterol, *HDL.C* high-density lipoprotein cholesterol, *LDL.C* low-density lipoprotein cholesterol, *PVD* portal vein diameter, *PVV* portal vein velocity

### Association of dual elastography parameters with histopathological changes in DILI patients

**Fig. 2:**
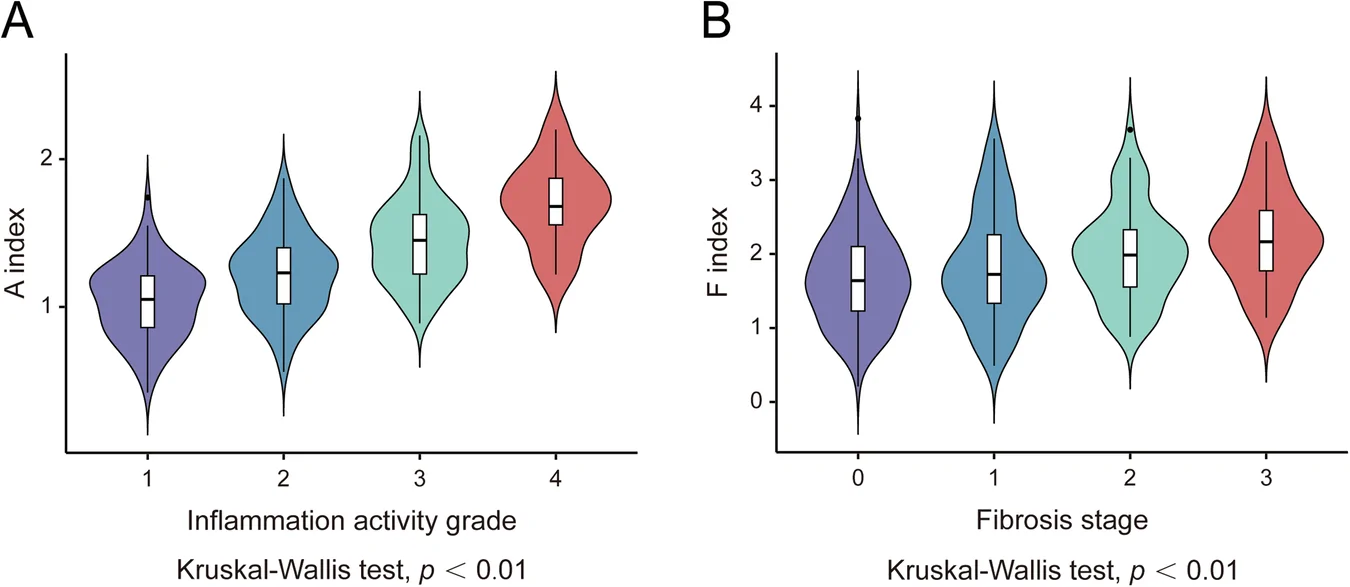
Evaluation of hepatic inflammatory activity and liver fibrosis at dual elastography. **A** Violin plot shows A index values increasing along with the progression of the inflammation activity grade. **B** Violin plot shows F index values increasing along with the progression of the hepatic fibrosis stage. A index, activity index; F index, fibrosis index

As the grade of hepatic inflammation increased, the dual elastography-derived A index values increased significantly (Fig. 2A and Supplementary Table S3). At the same time, the F index values also increased with the progression of hepatic fibrosis stages (Fig. 2B and Supplementary Table S4). The AUC, cutoff values, sensitivity, and specificity for both parameters are listed in Table 2. Specifically, for ≥ G2, the AUC for the A index was 0.791 (95% CI: 0.737–0.845), with sensitivity and specificity of 65.6% and 79.0%, respectively. For ≥ G3, the AUC was 0.790, with a sensitivity of 55.8% and specificity of 87.5%. For G4, the AUC was 0.853, with a sensitivity of 81.8% and specificity of 77.6%.

**Table 2:** Diagnosis performance of inflammation grade and fibrosis stage using A index and F index

	Cutoff	Prevalence	AUC (95% CI)	Sensitivity (%)	Specificity (%)
Inflammation grade					
A index					
≥ G2	1.225	224	0.791 (0.737–0.845)	65.6	79.0
≥ G3	1.435	129	0.79 (0.740–0.848)	55.8	87.5
G4	1.495	11	0.853 (0.75–0.955)	81.8	77.6
Fibrosis stage					
F index					
≥ S1	1.835	160	0.608 (0.545–0.671)	55.6	62.1
≥ S2	1.745	78	0.650 (0.582–0.718)	71.8	54.6
≥ S3	2.085	24	0.688 (0.585–0.792)	66.7	69.4
S4	1.705	4	0.651 (0.516–0.787)	100	46.5

*AUC* area under the curve, *CI* confidence interval, *A index* activity index, *F index* fibrosis index

### Construction and validation of a machine learning model to predict severe DILI using dual elastography parameters

#### Feature selection

The LASSO regression identified 10 features, which were combined with the two pre-defined core parameters (F index and A index). Collinearity analysis revealed that Vs was strongly correlated with the F index (r = 0.929). Consequently, Vs was excluded. The final model was built using 11 features: F index, A index, and the remaining nine LASSO-selected variables (BMI, INR, TB, ALB, HDL-C, COMP, SKEW, ASM, and ATT). The LASSO cross-validation profile and the correlation matrix are presented in Figs. S1 and S2, respectively.

#### Model construction and comparison

**Fig. 3:**
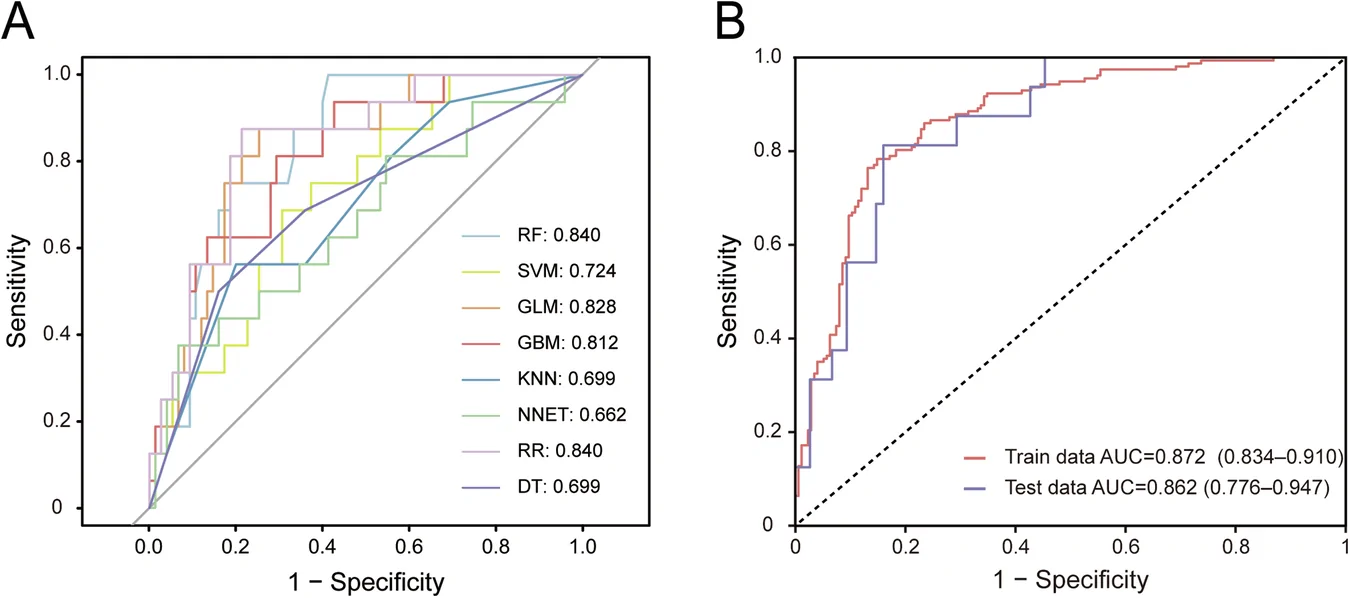
Receiver operating characteristic (ROC) curves. **A** Comparison of ROC curves across the eight candidate machine learning models. **B** ROC curves of the optimized regularized regression model on the training and test sets. RF, random forest; SVM, support vector machine; GLM, generalized linear model; GBM, gradient boosting machine; KNN, K-nearest neighbors; NNET, artificial neural network; RR, regularized regression; DT, decision tree

Eight ML models (RF, SVM, GLM, GBM, KNN, NNET, RR, and DT) were compared based on accuracy, precision, recall, F1-score, and AUC. Residual analysis and precision-recall curves are shown in Supplementary Fig. S3, and detailed performance differences are in Supplementary Table S5. As shown in Fig. 3A, the RR model achieved an AUC of 0.840, comparable to the RF model. However, the RR model demonstrated a substantially higher recall (0.875 vs. 0.438) and F1-score (0.596 vs. 0.452). Therefore, the RR model was selected for further optimization and analysis.

#### Model optimization and performance

The optimized RR model performed well in the training set, with an AUC of 0.872 (0.834–0.910), a sensitivity of 78.3%, and a specificity of 85.1%. On the internal test set, the model maintained a robust AUC of 0.862 (0.776–0.947), a sensitivity of 81.2%, and a specificity of 74.7% (Fig. 3B and Table 3). While the AUC was consistent, the predictive values shifted between the sets, with positive predictive value (PPV) decreasing from 82.6% to 40.6% and the negative predictive value (NPV) increasing from 81.4% to 94.9%. Detailed optimization metrics, including calibration curves and confusion matrices, are illustrated in Supplementary Fig. S4.

**Table 3:** Performance of the optimized regularized regression model

Metric	Training set	Test set	Interpretation
Accuracy	0.819 (0.779–0.860)	0.758 (0.670–0.846)	Overall correctly classified
Sensitivity	0.783 (0.717–0.848)	0.812 (0.605–1.000)	True positive rate (DILI detection)
Specificity	0.851 (0.799–0.905)	0.747 (0.651–0.844)	True negative rate (non-DILI)
PPV	0.826 (0.764–0.883)	0.406 (0.230–0.581)	Positive predictive value
NPV	0.814 (0.754–0.872)	0.949 (0.887–1.000)	Negative predictive value
F1-score	0.804 (0.751–0.849)	0.542 (0.344–0.705)	Harmonic mean of precision/recall
Balanced accuracy	0.817 (0.775–0.858)	0.780 (0.662–0.885)	Average of sensitivity/specificity
ROC AUC	0.872 (0.834–0.910)	0.862 (0.776–0.947)	Discriminative ability
PR-AUC	0.839 (0.768–0.893)	0.556 (0.293–0.776)	Precision-recall performance

*PPV* positive predictive value, *NPV* negative predictive value, *DILI* drug-induced liver injury, *ROC* receiver operating characteristic, *AUC* area under the curve, *PR* precision-recall

#### Model interpretation and visualization

**Fig. 4:**
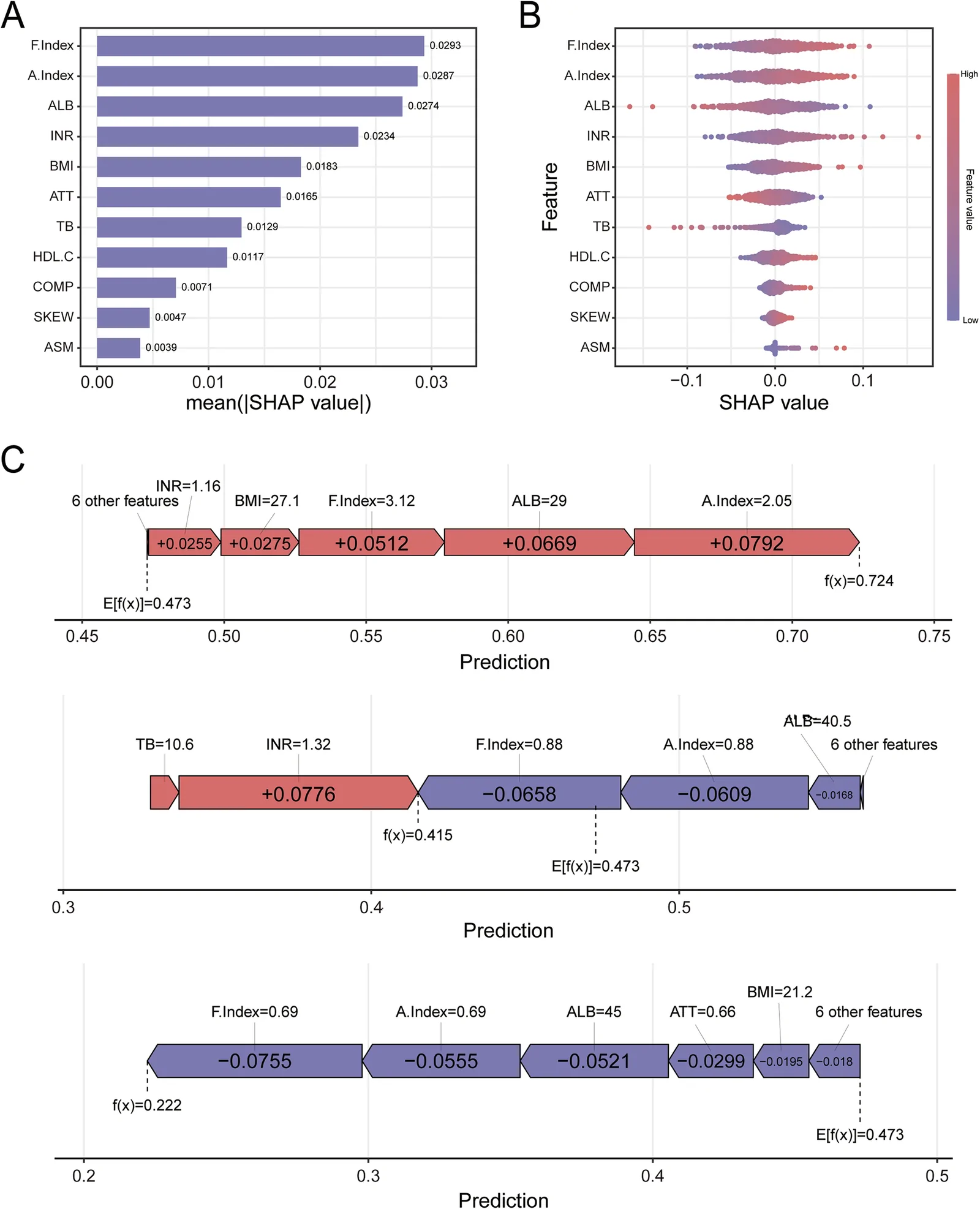
Interpretability of the machine model using SHAP. **A** SHAP summary bar plot. **B** SHAP beeswarm plot. **C** SHAP force plots for three representative patient samples (from top to bottom: high-risk, intermediate-risk, and low-risk). F index, fibrosis index; A index, activity index; ALB, albumin; INR, international normalized ratio; BMI, body mass index; ATT, acoustic attenuation coefficient; TB, total bilirubin; HDL.C, high-density lipoprotein cholesterol; COMP, complexity of low strain area within the region of interest; SKEW, skewness; ASM, angular second moment

SHAP analysis was used to interpret the RR model. Feature importance is ranked in Fig. 4A, and the beeswarm plot illustrates the direction of the impact (Fig. 4B). The analysis identified the F index, A index, and INR as factors associated with increased risk (higher values increased probability), whereas ALB and ATT were associated with decreased risk. Representative force plots (Fig. 4C) demonstrate feature contributions to individual predictions: elevated A and F indices combined with decreased ALB resulted in a probability of 0.724 in a high-risk patient, while the inverse pattern resulted in a probability of 0.222 in a low-risk patient. SHAP dependence plots confirmed that the probability of severe DILI increased with rising F index, A index, and INR and decreasing ALB levels (Supplementary Fig. S5).

### Development of an online web calculator for severe DILI based on the optimized RR model

The finalized optimized RR model was deployed as a publicly accessible web-based calculator (https://wznng666.shinyapps.io/RR55555/). This tool enables clinicians to input a patient’s clinical and dual elastography parameters to obtain a real-time estimated probability of severe DILI. Based on the optimal probability cutoff determined by the Youden index, the calculator provides automated risk stratification and offers preliminary management guidance. To ensure reproducibility, the source code for the model and the application is publicly available at https://github.com/zhuzhining-git/shiny.

## Discussion

To our knowledge, this is the first study to systematically apply dual elastography for the noninvasive assessment of histopathological severity in DILI. In a prospective multicenter cohort of 305 biopsy-confirmed patients, we demonstrated that the A index and F index correlate strongly with inflammation grade and fibrosis stage, respectively. A RR model integrating these dual elastography parameters with serological markers achieved robust discrimination for severe histological injury (AUC = 0.862 [95% CI: 0.776–0.947], accuracy = 75.8%), and SHAP analysis confirmed the contribution of the dual elastography indices to the model’s predictive output. Finally, the online DILI risk calculator developed in this study holds promise for facilitating clinical translation.

A notable finding was the dissociation between serum biochemistry and histological severity, as alanine aminotransferase (ALT) and aspartate aminotransferase (AST) did not differ significantly between the severe and non-severe groups [28], highlighting the limitations of conventional aminotransferases in reflecting structural damage in DILI [16, 29, 30]. This limitation extends to established clinical tools. In our cohort, neither MELD nor Hy’s Law demonstrated meaningful discriminative ability for severe histological injury, with AUCs approaching chance-level performance (AUC 0.563 and 0.506, respectively). DeLong tests confirmed that the RR model significantly outperformed both (*p* < 0.001) (Supplementary Fig. S6 and Table S6). This is clinically expected, as MELD was designed to predict mortality in end-stage liver disease and Hy’s Law to identify the risk of acute liver failure. These findings underscore the necessity of a dedicated predictive model for severe histological injury and risk stratification in DILI.

In dual elastography, the F index showed modest performance for early fibrosis, likely because increased stiffness is masked by concurrent inflammation and edema in acute DILI [18]. Although performance improved for advanced stages, cautious interpretation is warranted given the potential for low specificity. The texture heterogeneity parameters (SKEW and COMP) provided crucial microstructural insights. Severe DILI is characterized by increased SKEW and COMP, reflecting non-uniform strain distribution within the liver parenchyma. Mechanistically, this likely corresponds to the patchy pathology of DILI, where focal necrosis, inflammatory cell infiltration, and edema coexist with preserved hepatic lobules [31, 32], suggesting that RTE-captured heterogeneity is as informative as absolute stiffness. Additionally, SHAP analysis unexpectedly identified ATT as a protective factor despite its conventional association with steatosis [33, 34]. We speculate that acute hepatocyte necrosis and ballooning may alter tissue density, thereby decreasing sound attenuation [31, 35]. Furthermore, BMI emerged as a risk predictor, aligning with the “multiple hit” hypothesis where obesity-related subclinical inflammation lowers the threshold for hepatotoxicity [36, 37].

The clinical utility of this model lies primarily in risk stratification rather than standalone diagnosis. The high NPV (0.949) enables clinicians to exclude severe histological injury and avoid unnecessary liver biopsies in low-risk patients. Conversely, the moderate PPV (0.406) indicates that a high-risk prediction should serve as a warning signal warranting further evaluation, including confirmatory biopsy and considering corticosteroid therapy or artificial liver support. This NPV-PPV discrepancy stems from two factors. First, the low prevalence of severe DILI (18%), consistent with published epidemiological data, intrinsically inflates NPV while depressing PPV. Second, SMOTE oversampling, applied to address this imbalance, enhanced sensitivity (from 62.5% to 81.2%) at the cost of increasing false positives, which degraded calibration (Brier score 0.180 vs. 0.132; Supplementary Table S7) and lowering PPV [38]. Despite this trade-off, we retained the SMOTE-trained model to prioritize sensitivity, as missing a severe case risks irreversible progression to acute liver failure or chronic cirrhosis.

Nevertheless, this study has certain limitations. First, the sample size is small for machine learning. Despite regularization and similar training/test AUCs (0.872 vs. 0.862) suggesting controlled overfitting, limited positive cases drive class imbalance and constrained validation to internal splitting. The absence of an external cohort thus limits generalizability assessment, necessitating future validation in larger independent cohorts. Second, including patients meeting biopsy indications introduced selection bias, limiting accuracy in asymptomatic or mild DILI populations. Third, no DILI-specific pathology score exists. The adopted Scheuer system, designed primarily for viral hepatitis, may not fully capture DILI-specific features like eosinophil infiltration or cholestasis. Furthermore, the G + S ≥ 5 composite threshold defining severe DILI is an anatomical surrogate lacking prospective validation against hard clinical endpoints such as liver failure, transplantation, or death. Fourth, methodological aspects introduce potential bias, as outlier review remained operator-dependent despite IQR-based detection, and imputation assumptions remain a theoretical limitation despite low missingness (< 7%). Finally, although elastography occurred within 3 days pre-biopsy, temporal mismatch cannot be entirely excluded. Future studies should employ same-day acquisition protocols.

In summary, the dual elastography-derived F index and A index non-invasively assessed the hepatic inflammation and fibrosis in DILI. A machine learning model integrating these parameters with serological markers achieved robust discrimination and predictive capability for DILI patients with severe histological injury, substantially outperforming conventional tools. The accompanying online risk calculator provides preliminary guidance for clinical risk stratification and intervention. Given the distinct etiologies and DILI phenotypes in China compared to Western countries, future large-scale studies with external validation are necessary to confirm the generalizability of these findings.

## Supplementary information

Supplementary information

## Data Availability

The source code used for data analysis, model construction, and the development of the Shiny application in this study is publicly available in the GitHub repository: https://github.com/zhuzhining-git/shiny.

## Abbreviations

ALB: Albumin
ASM: Angular second moment
ATT: Acoustic attenuation coefficient
AUC: Area under the receiver operating characteristic curve
BMI: Body mass index
CI: Confidence interval
COMP: Complexity of low strain area within the region of interest
CV: Cross-validation
DILI: Drug-induced liver injury
DT: Decision tree
E: Young’s modulus
ENT: Entropy
F index: Fibrosis index
GBM: Gradient boosting machine
G: Inflammation activity grade
GLM: Generalized linear model
HDL.C: High-density lipoprotein cholesterol
IDM: Inverse difference moment
INR: International normalized ratio
IQR: Interquartile range
KNN: K-nearest neighbors
LASSO: Least absolute shrinkage and selection operator
MEAN: Mean relative strain value within the region of interest
MELD: Model for end-stage liver disease
NNET: Artificial neural network
NPV: Negative predictive value
PPV: Positive predictive value
PR-AUC: Area under the precision-recall curve
RF: Random Forest
ROC: Receiver operating characteristic
RR: Regularized regression
SD: Standard deviation
SHAP: SHapley Additive exPlanations
SKEW: Skewness
SMOTE: Synthetic minority oversampling technique
SWE: Shear wave elastography
SVM: Support vector machine
TB: Total bilirubin
Vs: Shear wave velocity
%AREA: Area of low strain within the region of interest
