# Supplementary material for: Noninvasive prediction of severe histopathology in drug-induced liver injury using a dual elastography-based machine learning model
Source: Eur Radiol. 2026 Jun 8;36(10):7609–20. doi: 10.1007/s00330-026-12644-y (PMC13574858; doi:10.1007/s00330-026-12644-y)
Supplement: Supplementary file 1 [file 330_2026_12644_MOESM1_ESM.pdf]

ELECTRONIC SUPPLEMENTARY MATERIAL

Non-invasive Prediction of Severe Histopathology in Drug-Induced Liver Injury Using Dual Elastography-Based Machine Learning Model

Supplementary information

Luping Qiu[1*], Liyun Xue[1*], Hui Feng[2*], Fankun Meng[3], Ying Zheng[3], Guangwen Cheng[1], Yao Zhang[4], Zhiyong Yin[4], Jing Wu[5], Jiabao Zhu[5], Jing Liang[1], Jie Yu[2#], Ping Liang[2#], Hong Ding[1#]

The detail of A index and F index

The A index and F index had been calculated in chronic liver disease of Japanese population using indices of strain imaging and Vs, and formulations were as follows:

F index = –12.259–0.0199 × MEAN –0.0069 × SD + 0.0068 × %AREA –0.0214 × COMP – 0.0772 × SKEW + 0.0862 × KURT + 1.6659 × ENT − 1.3422 × IDM + 42.1004 × ASM + 0.0011 × CON + 8.2076 × COR + 1.3869 × Vs;

A index = –7.408–0.0370 × MEAN – 0.0019 × SD – 0.0218 × %AREA –0.0059 × COMP + 0.1884 × SKEW – 0.2159 × KURT – 0.3597 × ENT – 5.1614 × IDM + 2.6551 × ASM + 0.0006 × CON + 15.1157 × COR + 0.5748 × Vs

Liver Fibrosis Index (LFI) had been calculated in Japanese patients with chronic viral hepatitis using strain imaging indices.

LFI = –0.009 * MEAN – 0.005 * SD + 0.023 *%AREA + 0.025 * COMP + 0.775 * SKEW – 0.281 * KURT + 2.083 * ENT + 3.042 * IDM + 39.979 * ASM – 5.542

Table S1. Hyperparameters of eight machine learning models.

Model	Optimal Hyperparameters	CV Accuracy
RF	mtry = 2, n.trees = 500	83.42%
SVM	C = 0.25, σ = 0.0683	83-84%
GLM	No required	86.73%
GBM	n.trees=100, depth=3, lr=0.1	86.72%
KNN	k = 9	82-83%
NNET	size=1, decay=0.1	83-86%
RR	α=0.1, λ=0.0021	83.90%
DT	cp = 0.0833	80-82%

Abbreviation: CV: cross-validation, RF: random forest, SVM: support vector machine, GLM: generalized linear model, GBM: gradient boosting machine, KNN: K-nearest neighbors, NNET: artificial neural network, RR: regularized regression, DT: decision tree.

Table S2. Baseline characteristics of the training and internal validation sets.

	Overall	Test Set	Training Set	*p*
n	305	91	214	
G+S≥5	55 (18.0)	16 (17.6)	39 (18.2)	1
BMI	23.04 [21.04, 25.00]	23.43 [21.74, 24.70]	22.90 [20.64, 25.18]	0.19
INR	1.04 [0.98, 1.11]	1.03 [0.97, 1.11]	1.04 [0.99, 1.11]	0.249
TB	23.40 [14.60, 50.90]	26.40 [15.70, 53.80]	21.85 [14.53, 48.25]	0.289
ALB	37.50 [34.30, 41.00]	37.90 [34.00, 40.05]	37.50 [34.50, 41.00]	0.647
HDL.C	0.97 [0.71, 1.26]	0.96 [0.70, 1.26]	0.97 [0.71, 1.29]	0.77
ATT	0.53 [0.46, 0.60]	0.53 [0.46, 0.62]	0.53 [0.46, 0.59]	0.459
F index	1.78 [1.33, 2.22]	1.80 [1.46, 2.29]	1.75 [1.27, 2.20]	0.389
A index	1.28 (0.33)	1.30 (0.33)	1.26 (0.33)	0.322
COMP	25.50 [21.80, 30.70]	25.40 [21.75, 30.90]	25.60 [21.83, 30.50]	0.704
SKEW	0.26 [0.13, 0.50]	0.29 [0.13, 0.51]	0.26 [0.13, 0.48]	0.804
ASM	0.00 [0.00, 0.00]	0.00 [0.00, 0.00]	0.00 [0.00, 0.00]	0.928

Abbreviations: BMI: body mass index, INR: international normalized ratio, TB: total bilirubin, ALB: albumin, HDL.C: high-density lipoprotein cholesterol, ATT: acoustic attenuation coefficient, F index: fibrosis index, A index: activity index, COMP: complexity of low strain area within the region of interest, SKEW: skewness, ASM: angular second moment.

Table S3. Evaluation of hepatic inflammatory activity grade at dual elastography.

Parameters	Inflammation activity grade				*p* value
	1	2	3	4	
n	81	95	118	11	
MEAN	111.00 [103.00, 115.00]	109.00 [102.12, 114.00]	106.50 [94.98, 113.00]	96.90 [94.50, 98.75]	<0.001
SD	57.36 (10.43)	59.06 (9.37)	61.46 (9.27)	66.79 (6.58)	0.002
%AREA	19.95 [11.90, 28.50]	21.10 [14.90, 29.40]	24.40 [15.05, 36.15]	34.30 [32.50, 36.70]	0.001
COMP	24.00 [20.80, 27.90]	24.50 [21.80, 28.35]	27.05 [22.68, 31.60]	33.50 [30.80, 38.30]	<0.001
KURT	2.42 [2.29, 2.62]	2.33 [2.25, 2.51]	2.42 [2.28, 2.59]	2.31 [2.20, 2.42]	0.099
SKEW	0.22 [0.12, 0.45]	0.23 [0.12, 0.37]	0.33 [0.16, 0.56]	0.49 [0.38, 0.57]	0.001
CONT	266.00 [217.00, 326.00]	304.00 [234.50, 355.00]	292.00 [235.00, 337.75]	388.00 [356.56, 442.00]	<0.001
ENT	3.70 [3.64, 3.72]	3.71 [3.67, 3.73]	3.68 [3.62, 3.72]	3.68 [3.63, 3.72]	0.008
IDM	0.09 [0.09, 0.10]	0.09 [0.09, 0.10]	0.10 [0.09, 0.12]	0.10 [0.09, 0.12]	0.044
ASM	0.00 [0.00, 0.00]	0.00 [0.00, 0.00]	0.00 [0.00, 0.00]	0.00 [0.00, 0.00]	0.255
CORR	0.96 [0.95, 0.96]	0.96 [0.95, 0.96]	0.96 [0.95, 0.97]	0.96 [0.95, 0.96]	0.169
ATT	0.56 [0.50, 0.64]	0.54 [0.48, 0.60]	0.49 [0.43, 0.56]	0.47 [0.37, 0.48]	<0.001
E	6.53 [5.25, 8.68]	9.51 [7.69, 12.34]	13.82 [10.50, 17.08]	18.67 [14.96, 20.62]	<0.001
Vs	1.48 [1.27, 1.70]	1.78 [1.60, 2.03]	2.14 [1.87, 2.39]	2.30 [2.06, 2.62]	<0.001
LFI	1.74 [1.35, 2.23]	1.90 [1.50, 2.46]	2.04 [1.54, 2.71]	2.69 [2.59, 2.73]	<0.001
F index	1.27 [0.93, 1.56]	1.70 [1.42, 2.08]	2.12 [1.81, 2.60]	2.69 [2.39, 3.16]	<0.001
A index	1.04 (0.25)	1.22 (0.27)	1.45 (0.29)	1.70 (0.28)	<0.001

Abbreviations: MEAN: mean relative strain value within the region of interest, SD: standard deviation, %AREA: area of low strain within the region of interest, COMP: complexity of low strain area within the region of interest, KURT: kurtosis, SKEW: skewness, CONT: contrast, ENT: entropy, IDM: inverse difference moment, ASM: angular second moment, CORR: correlation, ATT: acoustic attenuation coefficient, E: Young's modulus, Vs: shear wave velocity, LFI: liver fibrosis index, F index: fibrosis index, A index: activity index.

Table S4. Evaluation of hepatic fibrosis stage at dual elastography.

Parameters	Fibrosis Stage					p value
	0	1	2	3	4	
n	145	82	54	20	4	
MEAN	111.00 [101.00, 114.00]	109.00 [101.25, 114.00]	108.50 [98.75, 113.00]	100.50 [87.38, 106.00]	100.10 [92.43, 104.25]	0.011
SD	58.55 (9.31)	59.34 (10.44)	60.86 (9.46)	66.40 (7.66)	68.10 (8.31)	0.004
%AREA	21.30 [13.50, 30.30]	22.15 [15.25, 30.58]	21.50 [14.37, 33.05]	32.60 [24.67, 44.05]	31.85 [28.43, 38.48]	0.005
COMP	25.40 [21.70, 29.10]	25.15 [22.15, 29.72]	24.30 [21.42, 31.78]	30.10 [24.60, 34.95]	30.85 [28.10, 38.78]	0.042
KURT	2.39 [2.27, 2.55]	2.40 [2.28, 2.66]	2.37 [2.28, 2.57]	2.36 [2.25, 2.45]	2.20 [2.15, 2.28]	0.311
SKEW	0.23 [0.11, 0.43]	0.26 [0.13, 0.52]	0.34 [0.22, 0.50]	0.44 [0.18, 0.62]	0.34 [0.22, 0.50]	0.023
CONT	292.00 [235.00, 348.00]	286.50 [225.50, 335.50]	294.50 [223.25, 345.13]	317.00 [285.25, 356.50]	340.00 [320.00, 366.75]	0.138
ENT	3.70 [3.65, 3.73]	3.69 [3.62, 3.72]	3.69 [3.64, 3.72]	3.67 [3.54, 3.71]	3.70 [3.54, 3.73]	0.347
IDM	0.10 [0.09, 0.11]	0.09 [0.09, 0.11]	0.10 [0.09, 0.11]	0.11 [0.09, 0.14]	0.10 [0.09, 0.14]	0.329
ASM	0.00 [0.00, 0.00]	0.00 [0.00, 0.00]	0.00 [0.00, 0.00]	0.00 [0.00, 0.00]	0.00 [0.00, 0.01]	0.326
CORR	0.96 [0.95, 0.96]	0.96 [0.95, 0.96]	0.96 [0.95, 0.96]	0.96 [0.95, 0.97]	0.96 [0.96, 0.96]	0.517
ATT	0.54 [0.46, 0.60]	0.54 [0.46, 0.61]	0.52 [0.45, 0.60]	0.50 [0.43, 0.53]	0.62 [0.53, 0.69]	0.214
E	9.44 [6.32, 13.04]	9.68 [7.35, 14.54]	12.40 [8.60, 16.01]	14.37 [11.34, 18.49]	10.63 [9.18, 12.17]	0.001
Vs	1.76 [1.44, 2.08]	1.79 [1.56, 2.20]	2.04 [1.70, 2.31]	2.18 [1.94, 2.48]	1.88 [1.75, 2.02]	0.001
LFI	1.85 [1.41, 2.47]	1.89 [1.51, 2.50]	1.92 [1.51, 2.70]	2.56 [2.11, 2.98]	2.47 [2.24, 3.09]	0.012
F index	1.64 [1.23, 2.10]	1.73 [1.33, 2.26]	1.98 [1.56, 2.33]	2.20 [1.77, 2.67]	2.10 [1.99, 2.16]	0.001
A index	1.20 (0.31)	1.28 (0.34)	1.35 (0.30)	1.53 (0.32)	1.39 (0.26)	<0.001

Abbreviations: MEAN: mean relative strain value within the region of interest, SD: standard deviation, %AREA: area of low strain within the region of interest, COMP: complexity of low strain area within the region of interest, KURT: kurtosis, SKEW: skewness, CONT: contrast, ENT: entropy, IDM: inverse difference moment, ASM: angular second moment, CORR: correlation, ATT: acoustic attenuation coefficient, E: Young's modulus, Vs: shear wave velocity, LFI: liver fibrosis index, F index: fibrosis index, A index: activity index.

Table S5. Prediction performance of the eight machine learning models.

Dataset	Model	Recall	Precision	F1	Accuracy	AUC
Train	RF	1.000	1.000	1.000	1.000	1.000
Train	SVM	0.930	0.936	0.933	0.937	0.977
Train	GLM	0.860	0.804	0.831	0.834	0.889
Train	GBM	0.994	0.994	0.994	0.994	1.000
Train	KNN	0.975	0.832	0.897	0.895	0.980
Train	NNET	0.943	0.881	0.911	0.913	0.976
Train	RR	0.854	0.812	0.832	0.837	0.888
Train	DT	0.675	0.828	0.744	0.780	0.805
Test	RF	0.438	0.467	0.452	0.813	0.840
Test	SVM	0.313	0.278	0.294	0.736	0.724
Test	GLM	0.813	0.419	0.553	0.769	0.828
Test	GBM	0.563	0.529	0.545	0.835	0.813
Test	KNN	0.563	0.250	0.346	0.626	0.699
Test	NNET	0.438	0.304	0.359	0.725	0.662
Test	RR	0.875	0.452	0.596	0.791	0.840
Test	DT	0.500	0.400	0.444	0.780	0.699

Abbreviations: RF: random forest, SVM: support vector machine, GLM: generalized linear model, GBM: gradient boosting machine, KNN: K-nearest neighbors, NNET: artificial neural network, RR: regularized regression, DT: decision tree, AUC: area under the receiver operating characteristic curve.

Table S6. Predictive performance of the RR model, MELD score, and Hy’s Law for severe histological DILI.

Tool	AUC (95% CI)	Sensitivity	Specificity	p vs. RR model
RR model	0.862 (0.776–0.947)	81.2%	74.7%	—
MELD score	0.563 (0.486-0.639)	81.8%	42.8%	< 0.001
Hy's Law	0.506 (0.451-0.561)	83.6%	17.6%	< 0.001

Abbreviations: AUC: area under the receiver operating characteristic curve, CI: confidence interval, DILI: drug-induced liver injury, MELD: Model for End-Stage Liver Disease, RR: the predictive model developed in this study.

Table S7. Comparison of model performance with and without SMOTE for predicting severe histological liver injury.

Metric	With SMOTE (Train)	With SMOTE (Test)	Without SMOTE (Train)	Without SMOTE (Test)
AUC	0.872	0.862	0.841	0.872
Sensitivity	78.3%	81.2%	69.2%	62.5%
Specificity	85.1%	74.7%	89.1%	85.3%
NPV	81.4%	94.9%	92.9%	91.4%
Brier Score	0.186	0.180	0.125	0.132

Abbreviations: AUC: area under the receiver operating characteristic curve, NPV: negative predictive value.

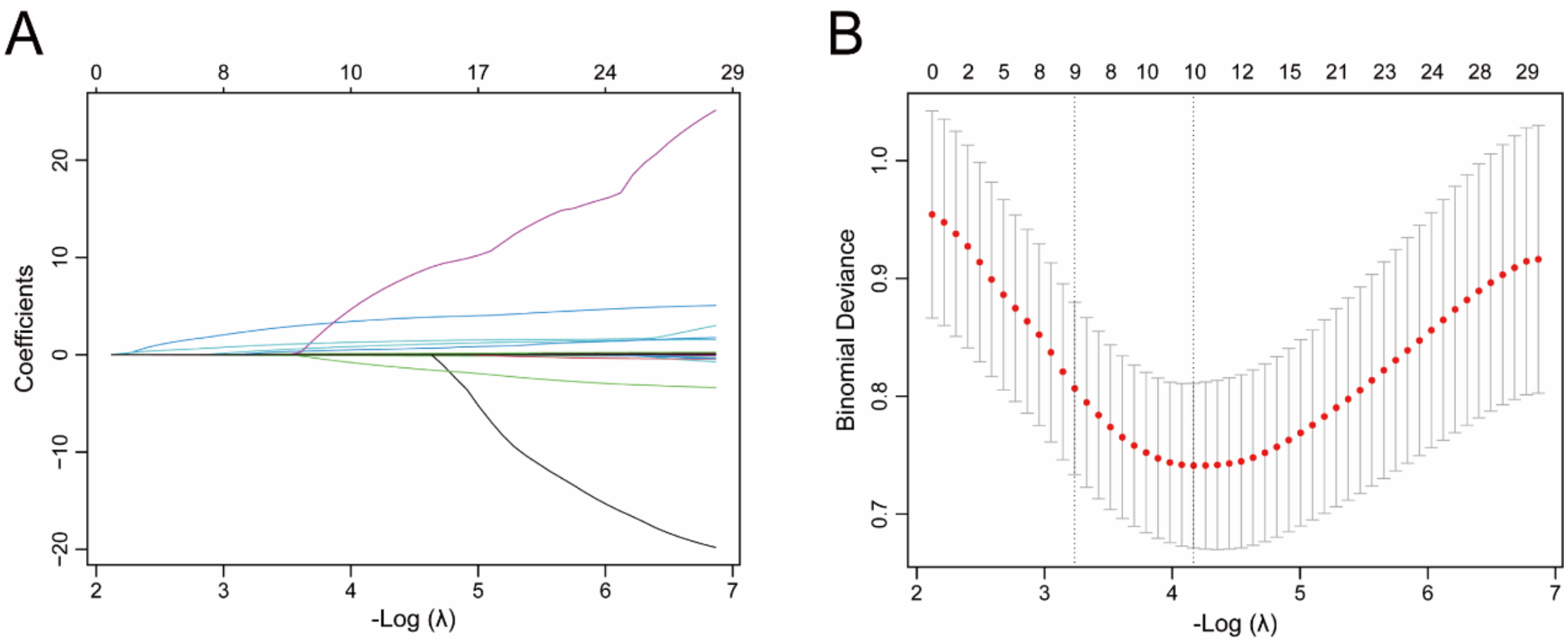

Figure S1. Selection of clinical features and dual elastography parameters using LASSO binary logistic regression. (A) LASSO coefficient path plot. (B) Cross-validation plot for LASSO regression.

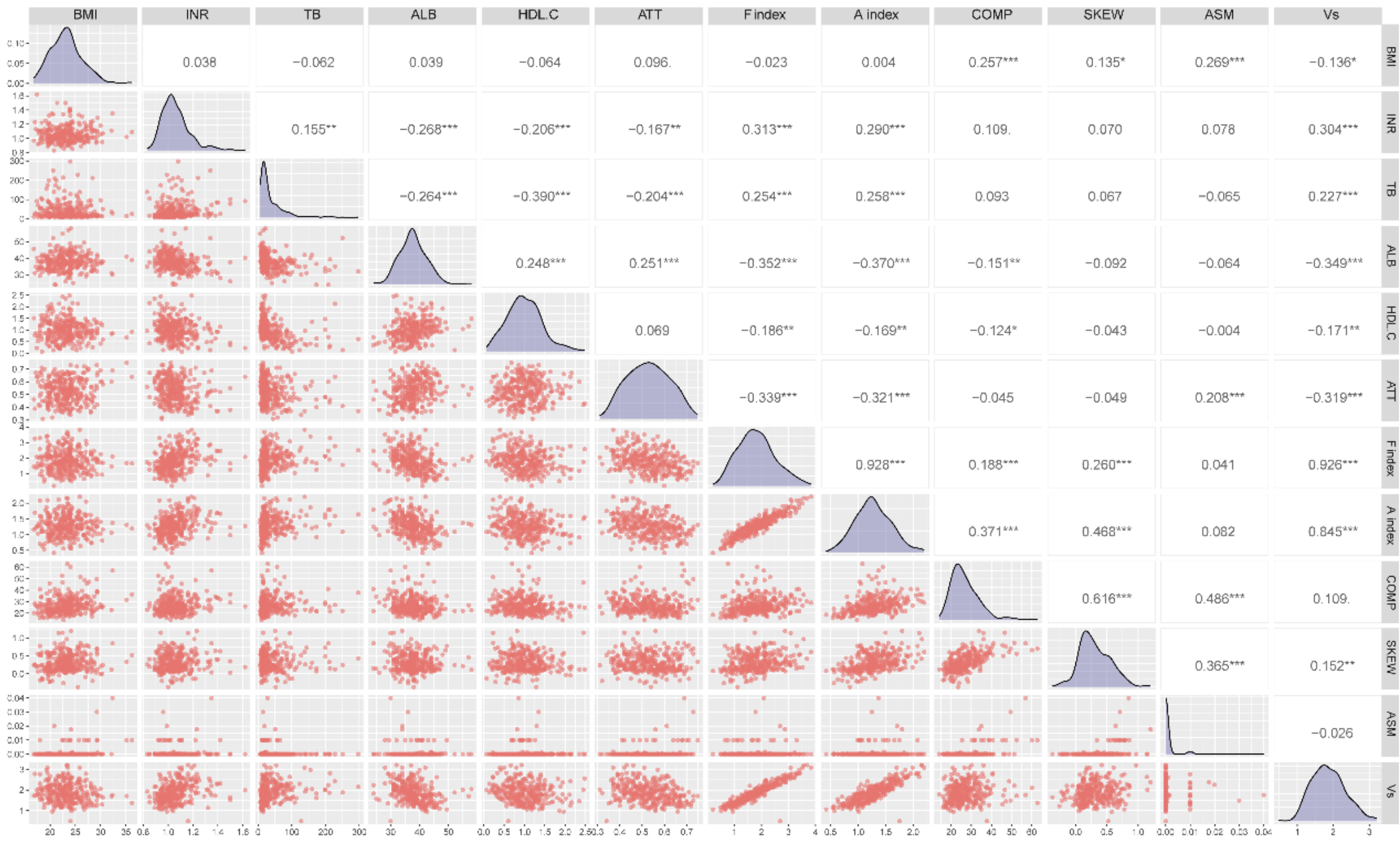

Figure S2. Correlation matrix of the 12 features selected for model construction.

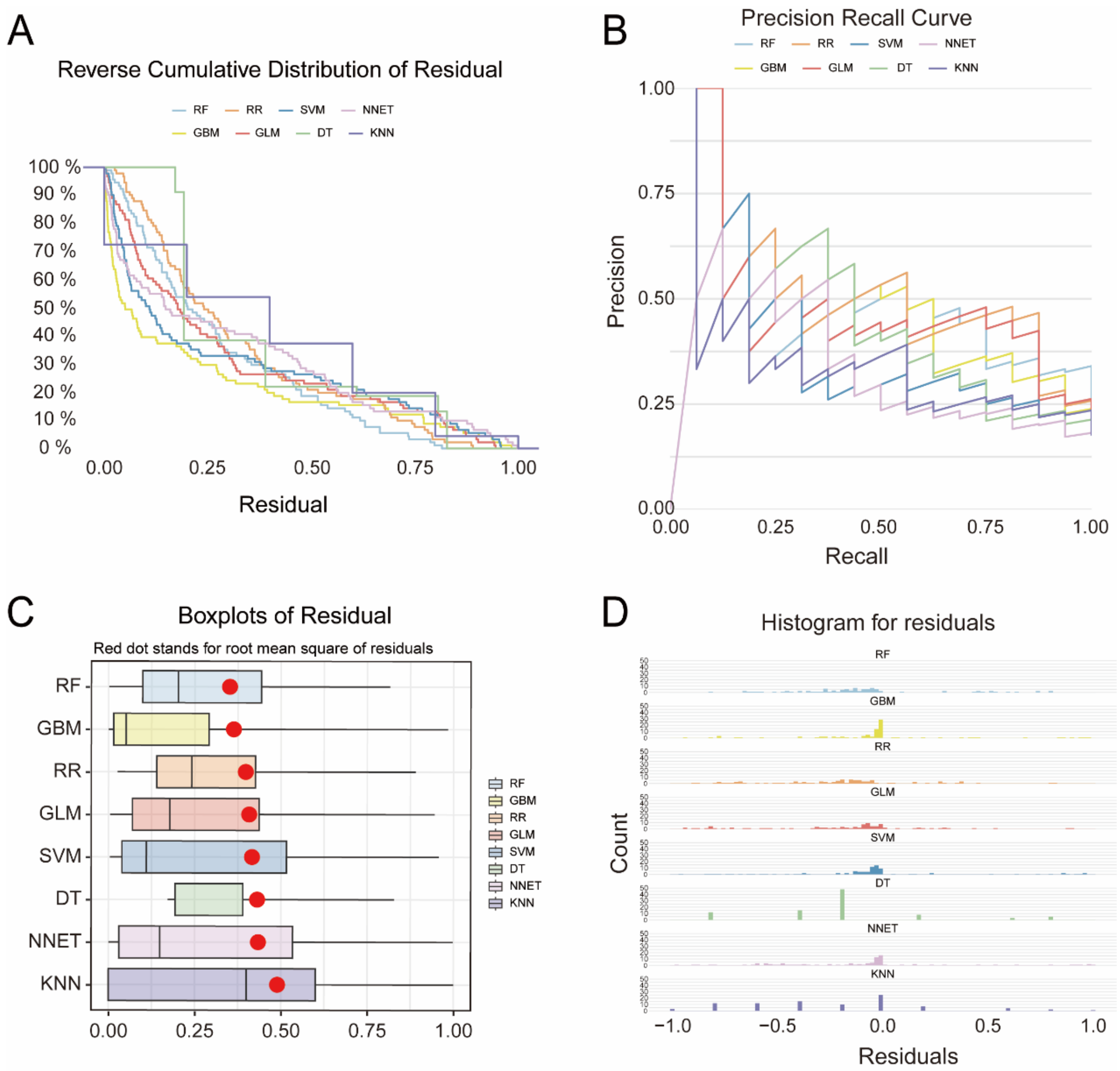

Figure S3. Model performance evaluation using DALEX residual analysis on the test set. (A) Reverse cumulative distribution curves of absolute residuals for eight machine learning models. (B) Precision-recall curves (PRC) for the eight models, demonstrating the precision-recall trade-off across varying probability thresholds. (C) Boxplots of absolute residuals for each model. (D) Histograms showing the distribution of absolute residuals for each model. Abbreviations: RR, Regularized Regression; RF, Random Forest; SVM, Support Vector Machine; GLM, Generalized Linear Model; GBM, Gradient Boosting Machine; KNN, K-Nearest Neighbors; NNET, Neural Network; DT, Decision Tree.

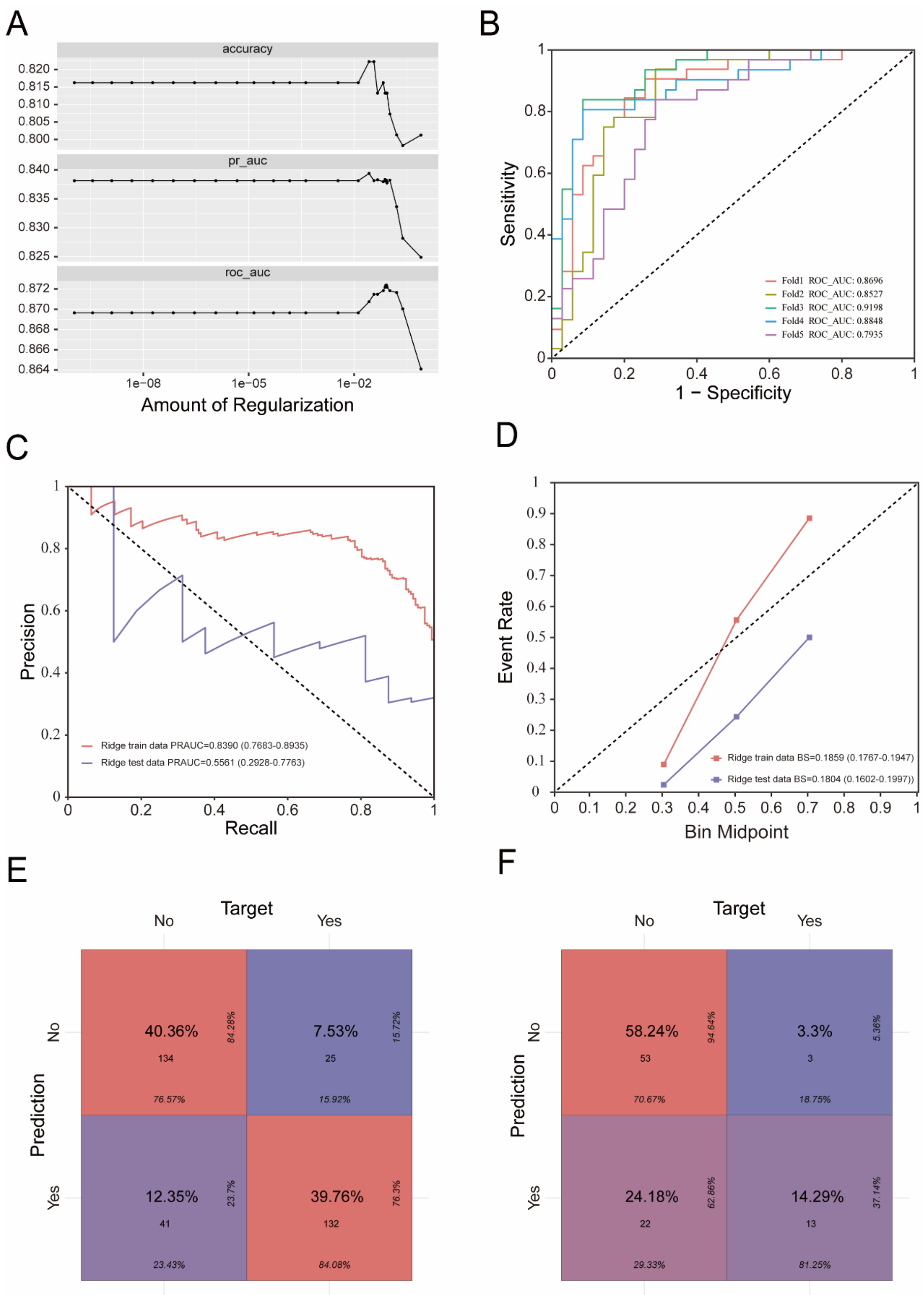

Figure S4. Model optimization and performance results. (A) Cross-validation performance curves for hyperparameter tuning. (B) ROC curves for the model across each fold of the 5-fold cross-validation. (C) PRC of the RR model. (D) Calibration curve of the RR model. (E) Confusion matrix for the training set. (F) Confusion matrix for the test set.

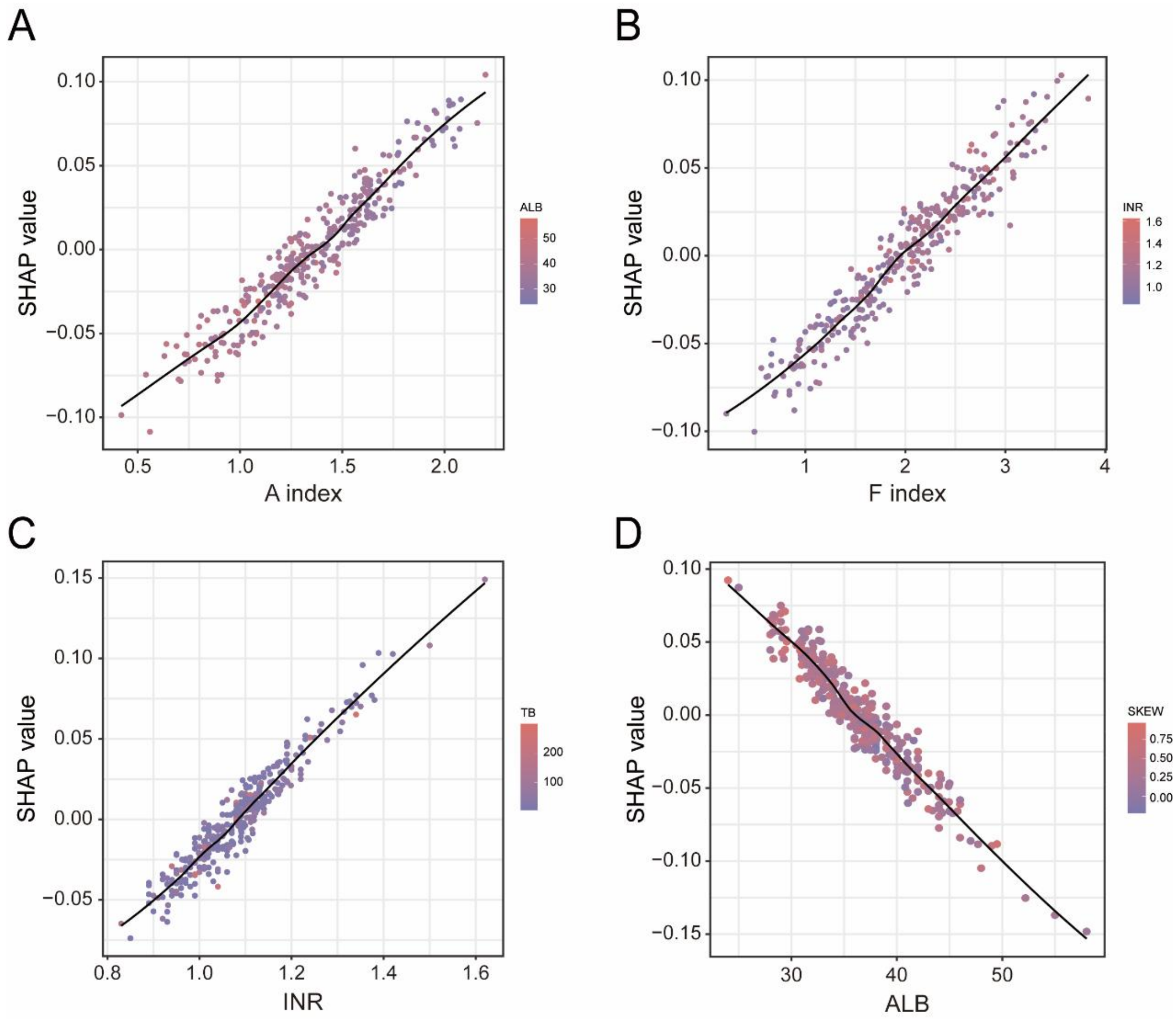

Figure S5. SHAP dependence plots illustrating the relationship between feature values and SHAP values. Each point represents a single sample, and the color of the point represents the magnitude of a second feature interacting with the feature on the x-axis. (A) A index, (B) F index, (C) INR, and (D) ALB.

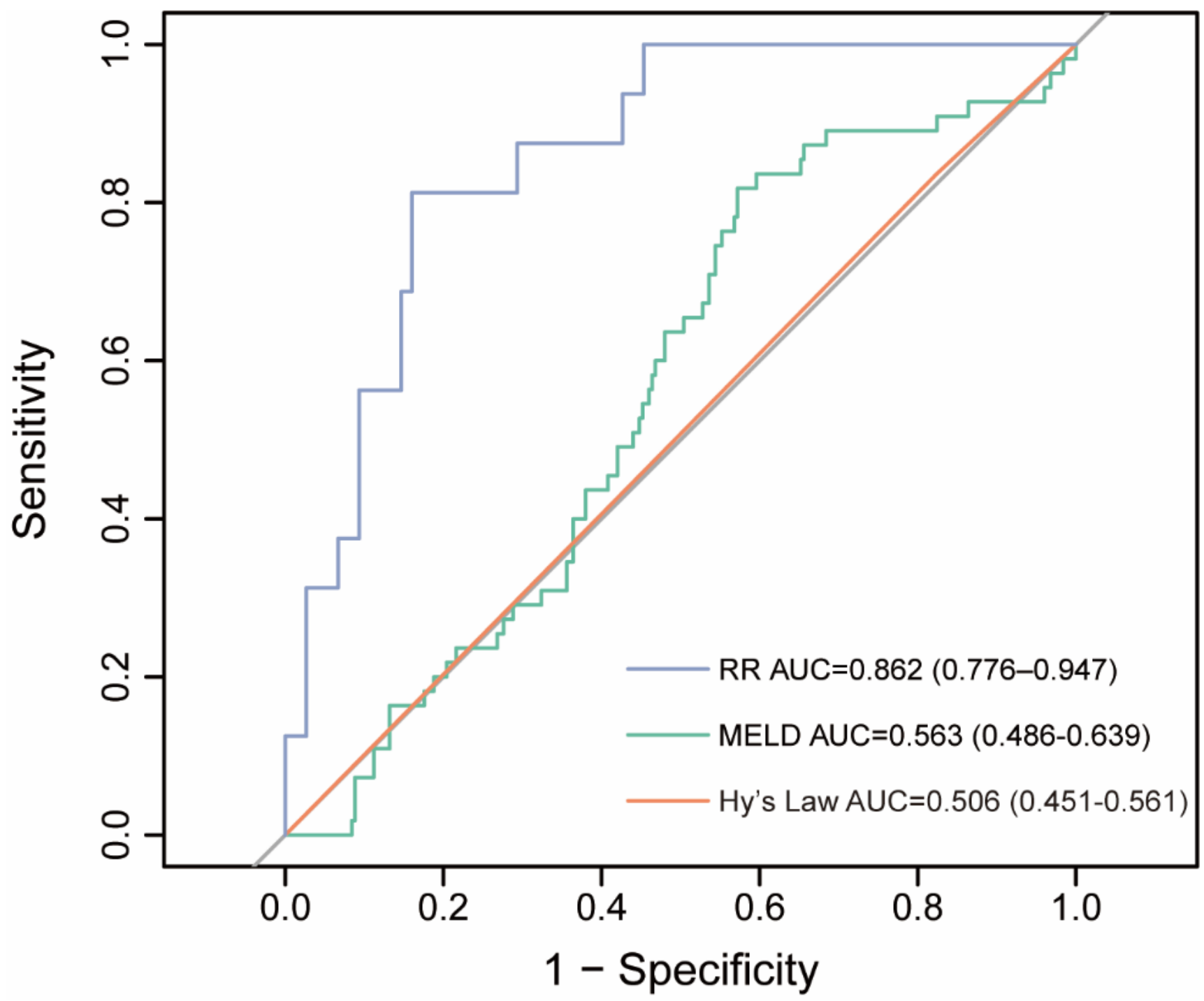

Figure S6. ROC curves of the RR model, MELD score, and Hy's Law for predicting severe histological DILI.
